# Supplementary material for: Developing health and environmental warning messages about red meat: An online experiment
Source: PLoS One. 2022 Jun 24;17(6):e0268121. doi: 10.1371/journal.pone.0268121 (PMC9231779; doi:10.1371/journal.pone.0268121)
Supplement: S1 Appendix — (DOCX) [file pone.0268121.s001.docx]

**S1 Appendix. Codebook.**

Summary of Number of items

| **Section** | **Number of items** |
| --- | --- |
| **Initial Prompts and Questions** | **3** |
| 1. **Health Warnings** | **26** |
| 1. **Climate Change Knowledge and Beliefs** | **2** |
| 1. **Climate Change Warnings** | **33** |
| 1. **Mini-experiment: Behavior Variant** | **1** |
| 1. **Mini-experiment: Roots of Warning Message** | **1** |
| 1. **E-cigarette items** | **10** |
| 1. **Demographics** | **11** |
| 1. **E-Closure** | **1** |
| **Total** | **53 for health (60 for climate change group)** |

**Estimated time to completion: 12 minutes (5 items/minute)**

| **# con**  **Variable name in Stata**  Variable description | **Item**  Question text  [note that all items will request (but not force, except where noted) a response] | **Respons**  **Response scale and note to programmers**  **Note for missing:** All missing values are coded as ‘99’. |
| --- | --- | --- |
| **meatfreq**  Red meat screener | **In the past 30 days, how often did you eat red meat?**  Red meat includes beef, lamb, pork, sausage, and ham. It also includes processed red meats such as bacon, hot dogs, and lunch meats. It **does not include** chicken, turkey, or seafood products.  [show image of red meats] | 0=Never  1=Less than 1 time per week  2=1 time per week  3=2-3 times per week  4=4-6 times per week  5=1 time per day  6=2 times per day  7=3 or more times per day |
|  | **I: Initial prompts and questions** |  |
| Consent | Consent form will be shown (attached with IRB as separate document). At the bottom of the consent form, it states “**By continuing with to the survey below, you acknowledge that you have read the information on this page and agree to be in this research study. Thank you!”** |  |
| I20  Prompt | In this survey, we will ask you some questions about red meat and tobacco. |  |
| I30  Instructions | [page break]  **Please read each question carefully. You will not be able to change your answers after you advance to the next page.** |  |
|  | **Main meat messaging experiment** |  |
|  | [Randomize people to one of 2 arms.]  Arm 1: Health warnings  Arm 2: Environmental warnings | **Note for programmers: Arm 1 should go to section A. Arm 2 should be redirected to section B.** |
|  | 1. **Health warnings** | **Note for programmers:** Show these messages to only the group randomized to receive the health warnings. |
| A10  Prompt  Grummon, Hall, Taillie, and Brewer (2019)  Warnings | [one-time prompt]:  **The next questions are about different messages about red meat.**  Red meat includes beef, lamb, pork, sausage, and ham. It also includes processed red meats such as bacon, hot dogs, and lunch meats. It **does not include** chicken, turkey, or seafood products.    [show image of red meats]  **You will look at 8 different messages and answer questions about each one.**  [Insert page break]  **Message list:**   1. Eating red meat increases your risk of type 2 diabetes 2. Eating red meat increases your risk of several types of cancer. 3. Eating red meat increases your risk of colon cancer. 4. Eating red meat increases your risk of colon cancer and rectal cancer. 5. Eating red meat increases your risk of cardiovascular disease. 6. Eating red meat increases your risk of heart damage. 7. Eating red meat increases your risk of stroke. 8. Eating red meat increases your risk of early death.   [under each message, program the below opening clause and then the individual PME items in a matrix]  **Please read the message above closely. Then answer the questions below.**  How much does this message... | Note for programmers: Show the prompt. Then, show the messages in random order, asking the questions below after each messages. |
| A20  PME: Discouragement  Baig et al. (2019)  Grummon et al. (2019)  **(Note: all discouragement items are labeled as the above warning followed by the letter ‘d’ (diabetesd, cancerd, colond, colonrectald, cvdd, heartd, stroked, deathd))** | discourage you from wanting to eat red meat? | 0=Not at all  1=Very little  2=Somewhat  3=Quite a bit  4=A great deal |
| A30  PME: Unpleasantness  Baig et al. (2019)  Grummon et al. (2019)  **(Note: all unpleasantness items are labeled as the above warning followed by the letter ‘u’ (diabetesu, canceru, colonu, colonrectalu, cvdu, heartu, strokeu, deathu))** | make eating red meat seem unpleasant to you? | 1=Not at all  2=Very little  3=Somewhat  4=Quite a bit  5=A great deal |
| A40  PME: Concern  Baig et al. (2019)  Grummon et al. (2019)  **(Note: all concern items are labeled as the above warning followed by the letter ‘c’ (diabetesc, cancerc, colonc, colonrectalc, cvdc, heartc, strokec, deathc))** | make you concerned about the health effects of eating red meat? | 1=Not at all  2=Very little  3=Somewhat  4=Quite a bit  5=A great deal |
|  | **Meat reduction questions** |  |
| A50  Ranking of items  **mosthealth** | [page break]  Which of these messages most discourages you from wanting to eat red meat?  **Click on an image to enlarge it.** | [answer choices are each 1 of the messages, in the same order as the messages were presented in the one by one messages ]  **Note to programmers:** Participants should choose only one option (not check all that apply) |
| A60  Intentions to limit meat consumption  Adapted from Malek et al (2019)  **reduceh** | How much do you plan to reduce your red meat consumption in the next 7 days? | 1=Not at all  2=Very little  3=Somewhat  4=Quite a bit  5=A great deal |
|  | 1. **Climate Change Knowledge and Beliefs** |  |
| B10. Belief in climate change  Benjamin et al (2016)  **climatebelief**  **humanbelief** | **Say how much you agree or disagree with the statements below.**  Climate change is occurring now.  The main cause of climate change is human activities. | **Note to programmers: Show two questions on different pages.**  1 = Strongly disagree  2 = Somewhat disagree  3 = Neither agree nor disagree  4 = Somewhat agree  5 = Strongly agree |
|  | 1. **Climate Change Messages** |  |
| C10  Prompt  Grummon, Hall, Taillie, and Brewer (2019) | [one-time prompt]:  **The next questions are about different messages about red meat.**  Red meat includes beef, lamb, pork, sausage, and ham. It also includes processed red meats such as bacon, hot dogs, and lunch meats. It **does not include** chicken, turkey, or seafood products.    **[Insert images of red meats]**  **You will look at 10 messages and answer questions about each one.**  [Insert page break]  **Message list:**   1. Eating red meat contributes to climate change. 2. Eating red meat contributes to climate change, which leads to extreme weather events. 3. Eating red meat contributes to global warming 4. Eating red meat increases your carbon footprint. 5. Eating red meat increases your greenhouse gas emissions. 6. Eating red meat contributes to water shortages. 7. Eating red meat increases water pollution. 8. Eating red meat increases deforestation. 9. Eating red meat harms the environment. 10. Eating red meat harms the planet.   [under each message, program the below opening clause and then the individual PME items in a matrix]  **Please read the message above closely. Then answer the questions below.**  How much does this message... | Note for programmers: Show the prompt. Then, show the messages in random order, asking the questions below after each messages. |
| C20  PME |  | **Note to programmers: Randomize the order of the PME items. All three items can appear on the same page.** |
| C30  PME: Discouragement  Baig et al. (2019)  Grummon et al. (2019)  **(Note: all discouragement items are labeled as the above warning followed by the letter ‘d’ (changed, weatherd, warmingd, carbond, gasd, shortaged, pollutiond, deforestationd, envd, planetd))** | discourage you from wanting to eat red meat? | 1=Not at all  2=Very little  3=Somewhat  4=Quite a bit  5=A great deal |
| C40  PME: Unpleasantness  Baig et al. (2019)  Grummon et al. (2019)  **(Note: all unpleasantness items are labeled as the above warning followed by the letter ‘u’ (changeu, weatheru, warmingu, carbonu, gasu, shortageu, pollutionu, deforestationu, envu, planetu))** | make eating red meat seem unpleasant to you? | 1=Not at all  2=Very little  3=Somewhat  4=Quite a bit  5=A great deal |
| C50  PME: Concern  Baig et al. (2019)  Grummon et al. (2019)  **(Note: all concern items are labeled as the above warning followed by the letter ‘c’ (changec, weatherc, warmingc, carbonc, gasc, shortagec, pollutionc, deforestationc, envc, planetc))** | make you concerned about the environmental effects of eating red meat? | 1=Not at all  2=Very little  3=Somewhat  4=Quite a bit  5=A great deal |
|  | **Meat reduction questions** | Show after participants have viewed all messages, answered PME items and the comprehension item. |
| C60  Ranking of items  **mostclimate** | [page break]  Which of these messages most discourages you from wanting to eat red meat?  **Click on an image to enlarge it.** | [answer choices are each 1 of the messages, in the same order as the messages were presented in the one by one messages ]  **Note to programmers:** Participants should choose only one option (not check all that apply) |
| C70  Intentions to limit meat consumption  Adapted from Malek et al (2019)  **reducec** | How much do you plan to reduce your red meat consumption in the next 7 days? | 1=Not at all  2=Very little  3=Somewhat  4=Quite a bit  5=A great deal |
| C80  Comprehension | [Randomize participant to see only one of the above climate change messages]  Was there anything confusing about this message? If so, please explain in the text box below.  **Click on the image to enlarge it.** | 1=No  2=Yes: [open ended] |

|  | 1. **Mini-experiment: Behavior variant** |  |
| --- | --- | --- |
| D10  Testing root messages | [page break]  **You will now look at a new message and answer a question about it.**  [Insert page break]  [Randomly assign participant to see only one of the below messages]:   1. Eating red meat contributes to species loss. 2. Eating too much red meat contributes to species loss. 3. Eating a lot of red meat contributes to species loss. 4. Consumption of red meat contributes to species loss. 5. Overconsumption of red meat contributes to species loss. 6. Excess consumption of red meat contributes to species loss. |  |
| D20  PME: Discouragement  Baig et al. (2019)  Grummon et al. (2019)  **eating**  **toomuch**  **lot**  **consumption**  **over**  **excess** | How much does this message discourage you from wanting to eat red meat? | **Note to programmers: Show this question on the same page as the randomized message.**  1=Not at all  2=Very little  3=Somewhat  4=Quite a bit  5=A great deal |
|  | 1. **Mini-experiment: Roots of warning messages** |  |
| E10.  Testing root messages | [page break]  **You will now look at a new message and answer a question about it.**  [Insert page break]  [Randomly assign participant to see one of the below messages]:   1. Eating red meat contributes to obesity. 2. Eating red meat may contribute to obesity. 3. Eating red meat is associated with obesity 4. Eating red meat increases your risk of obesity. 5. Eating red meat increases your chances of obesity. |  |
| E20.  PME: Discouragement  Baig et al. (2019)  Grummon et al. (2019)  **contribute**  **may**  **associate**  **risk**  **chances** | How much does this message discourage you from wanting to eat red meat? | **Note to programmers: Show the message on the same page as the PME question.**  1=Not at all  2=Very little  3=Somewhat  4=Quite a bit  5=A great deal |

|  | 1. **Cigarette items** | **Response** |
| --- | --- | --- |
| F10.  Prompt 1 | The next questions are about cigarettes. |  |
| F12.  Cig current use | Have you smoked at least 100 cigarettes in your entire life? | 1= Yes  0= No |
| F13.  Cig current use | Do you now smoke cigarettes every day, some days or not at all?  **[page break]** | 2= Every day  1= Some days  0= Not at all |
| F15.  Prompt 2 | Now we are going to show you a video advertisement and ask you some questions about it.  **[Randomize participant to intervention or control. If randomized to intervention arm: Randomize participant to 1 of 3 Real Cost smoking ads. If randomized to control arm: Show participant the 1 control ad]**  **[page break]** |  |
| F16.  Video | **SHOW AD**  **Click the play button if the video does not start automatically. After you watch the video, click the arrow to continue.**    **SHOW AD** | Intervention:  <https://www.youtube.com/watch?v=OoiryG6dJXY>  <https://www.youtube.com/watch?v=dnA-P0iMniA>  <https://www.youtube.com/watch?v=GKHq5WsASUM>    Control:  <https://vimeo.com/manage/395833921/general> |
|  | **[Randomize participant to items A20-A40 or A50-A70]** |  |
| F20.  Concerned_A | How much does this ad make you concerned about the health effects of smoking? | 1=Not at all  2=Very little  3=Somewhat  4=Quite a bit  5=A great deal |
| F30. Unpleasant_A | How much does this ad make smoking seem unpleasant to you? | 1=Not at all  2=Very little  3=Somewhat  4=Quite a bit  5=A great deal |
| F40.  Discourage_A | How much does this ad discourage you from wanting to smoke?  **[page break]** | 1=Not at all  2=Very little  3=Somewhat  4=Quite a bit  5=A great deal |
| F50  Concerned_B | Say how much you agree or disagree with these statements.  This ad makes me concerned about the health effects of smoking. | 1=Strongly disagree  2=Somewhat disagree  3=Neither agree nor disagree  4=Somewhat agree  5=Strongly agree |
| F60  Unpleasant_B | This ad makes smoking seem unpleasant to me. | 1=Strongly disagree  2=Somewhat disagree  3=Neither agree nor disagree  4=Somewhat agree  5=Strongly agree |
| F70  Discourage_B | This ad discourages me from wanting to smoke. | 1=Strongly disagree  2=Somewhat disagree  3=Neither agree nor disagree  4=Somewhat agree  5=Strongly agree |
| F75  Negative Affect | **[all participants get A75 on the same page as A20-A40 or A50-A70]**  How much did the ad make you feel scared?  **[page break]** | 1=Not at all  2=Very little  3=Somewhat  4=Quite a bit  5=A great deal |
| F80.  Feedback on control ad | Do you have any other comments you’d like to share about the ad you just watched? | [open ended] |

|  | 1. **Demographics** |  |
| --- | --- | --- |
| G0  Prompt | **The next questions are about you.** |  |
| G10  Gender  **gender** | How do you describe your gender identity? | 1=Male  2=Female  3=Nonbinary  4=Prefer to self-describe: [open text] |
| G20  Age  **age** | How old are you? Enter your age in years. | [Numerical entry]  [Limit from 18-99] |
| G20  Hispanic ethnicity  **hisp**  **hisptext** | Are you of Hispanic, Latino or Spanish origin? | 1=No, not of Hispanic, Latino, or Spanish origin  2=Yes, Mexican, Mexican American, Chicano  3=Yes, Cuban  4=Yes, another Hispanic, Latino, or Spanish origin (Enter Country of Origin) |
| G30  Race  **oldrace**  **texttotherrace** | What is your race? (check all that apply) | 1=White  2=Black or African American  3=American Indian or Alaska Native  4=Asian  5=Pacific Islander  6=Race not listed (please specify) |
| G40  Education  **educ** | What is the highest level of education you have completed? | 1=Less than high school or U.S. high school equivalent (GED)  2=High school diploma or U.S. high school equivalent (GED)  3=Associate or technical degree  4=4-year college degree |
| G50  Household Size  **house** | How many people are in your household, including you? | # of people [restricted to 1-20] |
| G60  Children in Household  **children** | How many children (ages 0-18) currently live in your household? | ____ [restricted to 0-15] |
| G70  Household income  **income** | Which of the following categories best describes your total household income in the last 12 months? | 1=Less than $10,000  2=$10,000 to $14,999  3=$15,000 to $24,999  4=$25,000 to $34,999  5=$35,000 to $49,999  6=$50,000 to $74,999  7=$75,000 to $99,999  8=$100,000 to $149,999  9=$150,000 to $199,999  10=$200,000 or more |
| G90  Political affiliation  **political** | Do you consider yourself to be: | **Note to programmers: Randomize the order of responses**  1=Liberal  2=Moderate  3=Conservative |
| G100  Neff et al (2019)  **comparemeat** | Compared to a year ago, the amount of red meat I eat now is: | 1=A lot less  2=Less  3=About the same  4=More  5=A lot more |
|  | 1. **Closure** |  |
| Closure | [page break] Anything you want to tell us about the study? Please leave your comments below. | [free text] |
| F20  Thank you code | Thank you for completing our survey! |  |

**Appendix: Example Warning Message Image (All warning messages have the same format, with the text in the above codebook inserted on a warning octagon)**


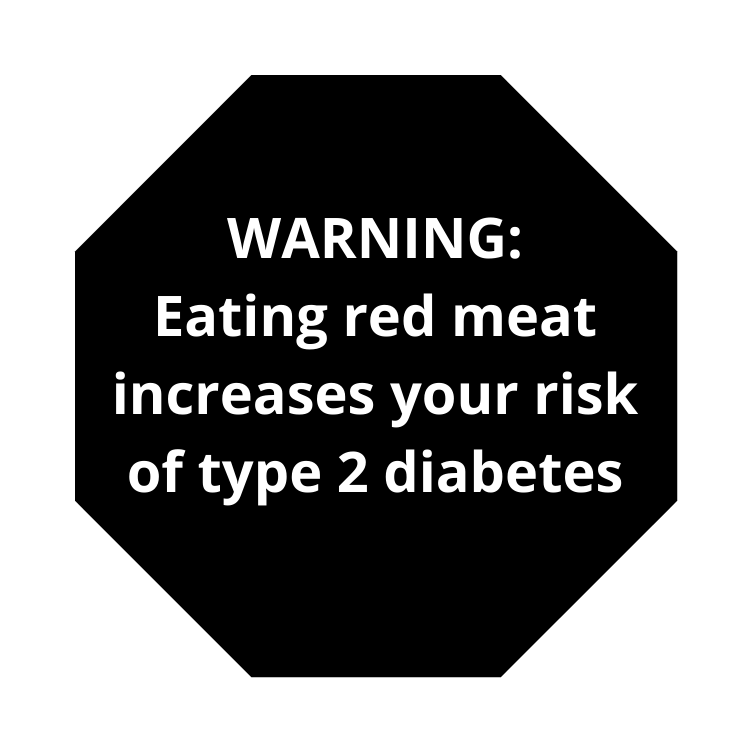


**Appendix: Image of Red Meats**


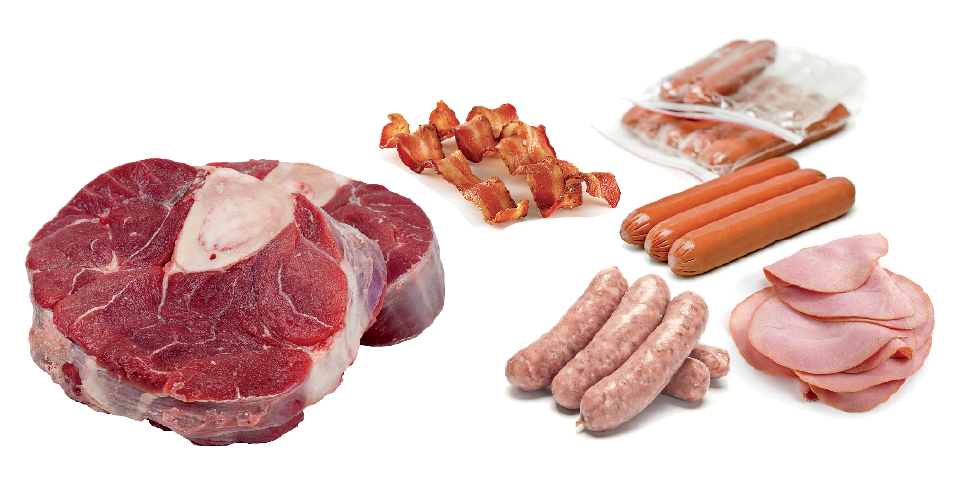


**References**

Baig, S. A., Noar, S. M., Gottfredson, N. C., Boynton, M. H., Ribisl, K. M., & Brewer, N. T. (2019). UNC perceived message effectiveness: validation of a brief scale. *Annals of Behavioral Medicine*, *53*(8), 732-742. [https://doi.org/10.1093/abm/kay080](https://academic.oup.com/abm/article-abstract/53/8/732/5131526?redirectedFrom=fulltext)

Benjamin, D., Por, H. H., & Budescu, D. (2017). Climate change versus global warming: Who is susceptible to the framing of climate change?. *Environment and Behavior*, *49*(7), 745-770. [https://doi.org/10.1177/0013916516664382](https://doi.org/10.1177%2F0013916516664382)

Centers for Disease Control and Prevention. (2013). National health and nutrition examination survey, 2009-2010 data documentation, codebook, and frequencies. *Dietary Screener Questionnaire*. Retrieved from: <https://epi.grants.cancer.gov/nhanes/dietscreen/questionnaires.html#web>

Chryst, B., Marlon, J., van der Linden, S., Leiserowitz, A., Maibach, E., & Roser-Renouf, C. (2018). Global warming’s “Six Americas Short Survey”: Audience segmentation of climate change views using a four question instrument. *Environmental Communication*, *12*(8), 1109-1122. <https://doi.org/10.1080/17524032.2018.1508047>

Grummon, A. H., Hall, M. G., Taillie, L. S., & Brewer, N. T. (2019). How should sugar-sweetened beverage health warnings be designed? A randomized experiment. *Preventive medicine*, *121*, 158-166. <https://doi.org/10.1016/j.ypmed.2019.02.010>

Neff, R. A., Edwards, D., Palmer, A., Ramsing, R., Righter, A., & Wolfson, J. (2018). Reducing meat consumption in the USA: a nationally representative survey of attitudes and behaviours. Public health nutrition, 21(10), 1835-1844.  <https://doi.org/10.1017/S1368980017004190>

Malek, L., Umberger, W. J., & Goddard, E. (2019). Committed vs. uncommitted meat eaters: understanding willingness to change protein consumption. *Appetite*, *138*, 115-126. <https://doi.org/10.1016/j.appet.2019.03.024>
