# Supplementary material for: Developing health and environmental warning messages about red meat: An online experiment
Source: PLoS One. 2022 Jun 24;17(6):e0268121. doi: 10.1371/journal.pone.0268121 (PMC9231779; doi:10.1371/journal.pone.0268121)
Supplement: S1 Table — *Five-point Likert scale ranging from 1 “Not at all” to 5 “A great deal” †Results from linear regression, adjusting for meat consumption as compared to a year ago. (DOCX) [file pone.0268121.s002.docx]

| **S1 Table. Adjusted perceived message effectiveness (PME)* and intention to reduce meat consumption in the next seven days* between Health and Environmental Messages** | | | |
| --- | --- | --- | --- |
| **Measure** | **Health Messages** | **Environmental Messages** | **p-value** |
|  | **Mean (SD)** | **Mean (SD)** |  |
| **PME** | 2.67 (1.12) | 2.25 (1.12) | <0.001 |
| **Intention to reduce meat consumption in the next 7 days** | 2.46 (1.14) | 2.18 (1.14) | <0.001 |
| *Five-point Likert scale ranging from 1 “Not at all” to 5 “A great deal”  †Results from linear regression, adjusting for meat consumption as compared to a year ago. | | | |
