# Supplementary material for: Developing health and environmental warning messages about red meat: An online experiment
Source: PLoS One. 2022 Jun 24;17(6):e0268121. doi: 10.1371/journal.pone.0268121 (PMC9231779; doi:10.1371/journal.pone.0268121)
Supplement: S2 Table — (DOCX) [file pone.0268121.s003.docx]

| **S2 Table. P-Values from Z-Tests comparing messages selected as most discouraging by U.S. adults randomized to the health (n=587) or environmental (n=584) group.** | | | | | | | |
| --- | --- | --- | --- | --- | --- | --- | --- |
|  |  |  |  | **p-value** |  |  |  |
| **HEALTH MESSAGES** | **Early death** | **Several types of cancer** | **Stroke** | **Colon cancer** | **Heart damage** | **Cardiovascular disease** | **Type 2 diabetes** |
| **Colon and rectal cancer** | <0.001 | 0.058 | 0.083 | <0.001 | 0.032 | 0.137 | <0.001 |
| **Early death** | -- | <0.001 | <0.001 | <0.001 | <0.001 | <0.001 | <0.001 |
| **Several types of cancer** | -- | -- | <0.001 | <0.001 | <0.001 | <0.001 | <0.001 |
| **Stroke** | -- | -- | -- | 0.013 | 0.680 | 0.804 | 0.099 |
| **Colon cancer** | -- | -- | -- | -- | 0.039 | 0.006 | 0.409 |
| **Heart damage** | -- | -- | -- | -- | -- | 0.509 | 0.216 |
| **Cardiovascular disease** | -- | -- | -- | -- | -- | -- | 0.058 |

|  | **p-value** | | | | | | | | |
| --- | --- | --- | --- | --- | --- | --- | --- | --- | --- |
| **ENVIRONMENTAL MESSAGES** | **Water pollution** | **Planet** | **Climate change-extreme weather** | **Deforestation** | **Global warming** | **Greenhouse gases** | **Climate change** | **Water shortages** | **Carbon footprint** |
| **Environment** | 0.116 | 0.408 | 0.185 | 0.562 | 0.016 | 0.013 | 0.005 | 0.247 | 0.116 |
| **Water pollution** | -- | 0.016 | 0.004 | 0.321 | 0.408 | 0.363 | 0.214 | 0.679 | 1.000 |
| **Planet** | -- | -- | 0.619 | 0.159 | 0.001 | <0.001 | <0.001 | 0.047 | 0.016 |
| **Climate change – extreme weather** | -- | -- | -- | 0.057 | <0.001 | <0.001 | <0.001 | 0.013 | 0.004 |
| **Deforestation** | -- | -- | -- | -- | 0.069 | 0.057 | 0.025 | 0.562 | 0.321 |
| **Global warming** | -- | -- | -- | -- | -- | 0.934 | 0.679 | 0.214 | 0.408 |
| **Greenhouse gases** | -- | -- | -- | -- | -- | -- | 0.741 | 0.185 | 0.363 |
| **Climate change** | -- | -- | -- | -- | -- | -- | -- | 0.098 | 0.214 |
| **Water shortages** | -- | -- | -- | -- | -- | -- | -- | -- | 0.679 |
