# Supplementary material for: Developing health and environmental warning messages about red meat: An online experiment
Source: PLoS One. 2022 Jun 24;17(6):e0268121. doi: 10.1371/journal.pone.0268121 (PMC9231779; doi:10.1371/journal.pone.0268121)
Supplement: Attachment — Submitted filename: PLOS One Revision Response Letter_FINAL.docx [file pone.0268121.s004.docx]

January 6^th^, 2022

Dr. Emily Chenette
Editor-in-Chief

PLOS One

Dear Editor,

We thank you and the reviewers for your comments on our recently submitted manuscript titled “Developing health and environmental warning messages about red meat: An online experiment” (ID PONE-D-21-09001). We appreciate the thoughtful comments from the reviewers and believe the revised manuscript is significantly stronger for having incorporated their suggestions.

We are attaching a revised version of the manuscript with track changes. We are also attaching a table to this letter that contains point-by-point responses to the reviewers’ comments.

Another change is that co-author Christina Chauvenet has requested to be moved from the author list to the acknowledgements section as due to a job change, she can no longer to contribute to reviewing or contributing to the manuscript. We have also added Carmen Prestemon as a co-author. Carmen is a research assistant who contributed to the project during the initial phases and also contributed writing, editing, and analysis during the revision; we believe that her contributions warrant authorship.

We look forward to hearing from you regarding our submission. We would be happy to respond to any further questions and comments that you might have.

Kind regards,

Lindsey Smith Taillie, PhD

Assistant Professor, Department of Nutrition 
University of North Carolina at Chapel Hill

**Ms. Ref. No.: ID** PONE-D-21-09001

**Title: “**Developing health and environmental warning messages about red meat: An online experiment”

| **#** | **Reviewers’ comments** | **Revision or Response** | **Line Number** |
| --- | --- | --- | --- |
|  | **Editor** |  |  |
| **1** | This is more of a reject with opportunity to resubmit than a major revision. Revision doesn't guarantee acceptance and both reviewers had concerns. Please thoroughly respond to all reviewer comments. Please pay close attention to R1's statistical analyses concerns. Please also provide power calculations for the trial and if primary are powered please further clarify for secondary within entire manuscript (i.e. exploratory). If not prospectively powered, clarify in abstract and throughout manuscript pilot study or exploratory. With R2, please perform a sensitivity analyses pre vs. COVID-19 to see if it affects results. | Thank you for this opportunity. We did not conduct a formal power calculation, as this was an exploratory study. The goal of the study was to develop and test messages to identify the most effective health and environmental messages, which would then be tested in a subsequent behavioral trial. We have modified language throughout the abstract, and manuscript to indicate that this was an exploratory study focused on message development and to more clearly state the study goals. In addition, we added a sentence to the Methods that states: “No *a priori* power calculations were conducted for this exploratory study.”  In regard to Reviewer 2’s comment, we examined the data more closely and determined that all data were collected within a 4-day period in late March 2020. Given this, we determined that this was not a sufficient time frame to conduct sensitivity analyses. We now have included the dates of the data collection in the MS so that readers can see this also (March 22-25, 2020). | Lines 166-167 |
|  | **Reviewer #1** |  |  |
| **2** | This study investigates the effectiveness of health and environmental warning messages on consumers’ intention to reduce red meat consumption via an online experiment. The authors claim that health warning is more effective than environmental messages. The topic is timely and relevant. However, the measurement of the effectiveness is poorly designed. Based on the results resented by far, I am far from convinced that the health warning messages are more effective. | Thank you for this useful comment. We appreciate this concern. This comment helped us realize that the purpose and outcome of this study may not be clear. This study does not evaluate the *effectiveness* of health or environment messages, as it does not evaluate the impact of these messages on behavior. Rather, it evaluates perceived message effectiveness (PME), or how much the participants think that the message will discourage consumption, make consumption unpleasant, or make them feel concerned about health or environmental effects. Perceived message effectiveness is a commonly used measure in message development because it is predictive as to messages’ potential to change behavior and is sensitive to subtle differences between messages. PME is therefore useful for testing a variety of messages against one another before deploying the most promising messages in larger effectiveness trials. Although research indicates that PME is predictive of behavior change, we agree with the reviewer that the actual effectiveness of these messages on behavior requires further study. We have added text to clarify this point throughout the paper so that the reader knows that this was not an effectiveness trial but rather a preliminary study to develop messages that would later be tested in an actual effectiveness trial. For example, in the introduction of the abstract, we now state that this study developed messages and explored participants’ reactions to them (Lines 25-28):  “Warning messages are effective for reducing purchases of products like sugary beverages but have not been developed for red meat. This study developed health and environmental warning messages about red meat and explored participants’ reactions to these messages.”  In the conclusion of the abstract, we call for future research to evaluate effectiveness (Line 46):  “Future studies should measure the impact of these messages on behavioral outcomes.”  Similarly, in the introduction, we now note that no studies have *developed* warning messages (Lines 96-97):  “Prior studies suggest the promise of warning messages [1, 2], but studies have not yet developed warning messages about the environmental impact of red meat more specifically.”  We have amended the objective to clearly state that a major goal of this study was message development and initial evaluation, with the goal of identifying which messages should be evaluated in future studies of effectiveness (Lines 106-109):  “The objective of this study was to develop health and environmental warning messages about red meat consumption and examine U.S. consumers’ reactions to these messages, with the overarching goal of identifying which messages hold the most promise for use in future studies to test behavioral impact.” | Lines 25-28  Line 46  Lines 96-97  Lines 106-109 |
| **3** | The most important concern that I have for this study is the choice of the effectiveness measurement. In the current version, the authors use perceived message effectiveness (PME) as the key measurement. PME is a self-report Likert scale about the message, e.g., I think this message discourage me from wanting to eat red meat a great deal or not at all.  If we assume that the message works via the following mechanism: (a)messages --> (b) change thoughts --> (c) change behavior (e.g., consumption frequency). What the authors measured is only the first step (from a to b) - the impacts of message on thoughts not behavior. Given that there are field studies already successfully measured the impacts of nutrition education on direct consumption outcomes, I find that a study only investigating the partial impacts of the message on people mind could provide limited solid and novel insights to the existing literature.  Further, a large amount literature has demonstrated that the hypothetical bias associated with self-report behavior intention, and such bias can be particularly large when social desirability bias exists. I believe both health and environmental message could induce social desirability bias which could exacerbate hypothetical bias. | We appreciate the opportunity to clarify the benefits of studying PME as a primary outcome. Our use of PME was guided by a large literature using this measure to evaluate warning messages at the early stages of message development. To summarize, we used PME as our primary outcome because it is sensitive to small differences in messages, is predictive of behavioral change, is commonly used in other studies of message development, and reflects an important step on the proposed conceptual pathway of how warnings lead to behavioral change.  To explain in more detail, first, as noted above, PME is useful for message development studies because meta-analyses indicate that it is predictive of behavior change and because it is sensitive to differences between messages. We have added text to the paper to better justify our choice of primary outcome for this message development study, including citations of prior studies that have used this same measure (Lines 110-114):  “Our primary outcome was perceived message effectiveness (PME), a measure that has been used extensively in similar experiments to identify the potential impact of food and beverage warning labels on consumers [2-7]. PME is a measure that is sensitive enough to detect small differences between warnings, yet is also predictive as to messages’ ability to change actual behaviors.”  Second, as noted before, the goal of this research was *message development* as the first part of a larger study to test the effect of warning labels on actual behavior. Prior to conducting a trial of warnings’ effects on behavior, it is necessary to determine which of many potential messages hold the most promise for being effective at changing behavior. Our prior studies of sugary drink warnings have found that subtle differences in message design (e.g., whether the message includes the word “WARNING” or whether it is displayed on a rectangle vs. octagon-shaped label) can elicit significant differences in consumer responses to messages [2]. This highlights the importance of testing different designs before deploying messages in large-scale trials. In turn, simulation modeling studies indicate that even small differences in warnings’ effectiveness at changing behavior can yield substantial differences in warnings’ impacts on population health outcomes such as obesity [8]. Message development studies are therefore a critical first step toward designing interventions that maximize population health benefits.  When evaluating multiple messages, a sensitive outcome like PME is necessary to be able to detect small differences between promising messages. We have revised the manuscript to make clear that our goal was message development (Lines 106-109):  “The objective of this study was to develop health and environmental warning messages about red meat consumption and examine US consumers’ reactions to these messages, with the overarching goal of identifying which messages hold the most promise for use in future studies to test behavioral impact.”  In addition, PME fits in with the proposed conceptual model of how food and beverage warnings lead to behavioral change [9]. Second, a review of PME that included 75 studies conducted in 21 countries with more than 61,000 participants demonstrated a wide use of the scale in message development studies [10]. Third, the PME measure we used has been found to be predictive of behavior and therefore is a good predictor of the likely effectiveness of messages [11].  Although we believe PME fits the goals of this study, we agree with the reviewer that the next logical step is to assess whether these messages influence behavioral outcomes. Therefore, we have added text to the discussion to indicate that assessing PME was a limitation, because it is not a behavioral measure. The text now reads (Lines 507-512):  “Additional limitations include that this study assessed only perceived effectiveness and did not assess behavioral outcomes, such as purchases of red meat, although previous research on communication campaigns has found that PME, our primary outcome, is predictive of behavioral change [12]. Further research will clarify how consumers alter food purchases in response to red meat warnings.” | Lines 106-114  Lines 507-512 |
| **4** | In term of statistical analysis, I strongly disagree that authors directly calculated means of Liker scale, e.g., 5= a great deal and 1= not at all, and the average is 2.6. By taking average of those scales, the authors are assuming the distance between 1=not at all and 2 = somewhat not important are equal to the distance between 4=somewhat important and 5=a great deal, which is a very poor assumption and is also not the common practice. Corresponding to that, instead of using linear regression model (table 5), the author should use ordered logit model. | The UNC PME is a scale which represents the average of three PME items. Because this scale is continuous, we have analyzed it using linear regression, similar to our prior studies using this scale [2-7]. We do not believe that an ordered logit model is appropriate because the average of the three items is a continuous, not categorical, variable.  We also note that using linear regression to analyze scales as continuous measures is common practice in many fields, including in health behavior [13]. Using linear regression to analyze scales is appropriate for our study goals for several reasons.  First, the results from linear regression (e.g., means) are often easier for readers to interpret than results from ordered logit models. Second, a recent review paper [13] described several conceptual and empirical reasons why banning the use of parametric analyses (such as linear regression) for aggregated scale data such as ours may be overly restrictive. Although scholars have argued against using linear regression for scale scores because this approach assumes that the distance between each response option is equivalent, several previous studies find that this assumption often holds. For example, Harpe et al. describe studies that examined the effect of changing measurement levels or modifying the distance between categories in ordinal measures [13]. These studies found that these differences are relatively unimportant important and that parametric approaches are acceptable for analyzing aggregated scale data even when the data are not strictly on the interval level of measurement [14-18]. In addition, many of the methods used to develop and validate these scales (e.g., factor analysis and item response theory), including the methods used to develop the PME scale [19], are based on parametric statistics and use categorical variables to create a measure of an underlying latent variable, which are considered continuous variables [20, 21].  For these reasons, we believe our use of linear regression to analyze PME is appropriate. If the editor feels strongly that additional analyses using another approach are warranted, we are happy to carry those out. | N/A |
| **5** | Also, I strongly disagree with the author that, because messages are randomly assigned, the balance tests are therefore not required. Balance tests are essential to demonstrate that your randomization was successful. Unsuccessful randomization is fairly common even when researchers thought they randomly assigned subjects to treatment groups. | Thank you for this comment. We did not conduct balance tests based on CONSORT guidance for RCTs [22]. According to CONSORT, the decision to control for certain covariates is better guided by theory and prior empirical evidence than by the specifics of an individual trial’s data. Any imbalances in participant characteristics between groups in a randomized trial would be, by definition, due to chance, and so would not be confounders (as they would be unrelated to the exposure variable because that variable was randomly assigned). Significance testing to look for imbalances is bound to identify characteristics that are not balanced due to chance and therefore is not recommended by CONSORT. Given this, it is recommended to select variables a priori that are most likely to be confounders, rather than base this decision off of statistical testing [22].  Nonetheless, we understand the desire to compare participant characteristics by treatment arm. For that reason, and based on this request, we have conducted balance tests and report those results in an additional column in **Table 3.** We found that one variable was unbalanced by chance (change in red meat consumption), and so we ran a sensitivity analysis in which we used linear regression controlling for this variable and reported those results in **S1 Table.** Since the results were very similar to the unadjusted analysis (now reported in **Table 4**), we retained the unadjusted analysis for our main presentation of results, as noted in our study pre-registration. To the manuscript we added:  “We used chi-square tests to check that socio-demographic characteristics were balanced across arms (**Table 3**). Of the nine balance tests conducted, 1 was significant: participants randomly assigned to the environmental arm were slightly less likely to report consuming less meat as compared to a year ago (24.8%) relative to participants randomly assigned to the health warning arm (23.4%) (*p*=0.04).” | Lines 240-244 |
| **6** | For the experiment design, shouldn’t there be a baseline group where they received a placebo information so that their attitude can be used as the gold standard benchmark? | We agree that a control group that receives placebo information is a useful approach for evaluating the relative impact of a new message (or warning) compared to receiving neutral information. However, the main purpose of this study was to understand whether health or environmental warnings were better relative to each other (to inform our selection of which label type to use in our subsequent behavioral trial). We therefore decided to omit a control group so we could maximize power for comparing the two warning types to one another  In addition, there is precedent for this decision to omit a control group during message development studies, as we have successfully done this for studies developing and evaluating sugar-sweetened beverages [23].  We have added this explanation to the methods section where the choice of study arms is described:  “Similar to previous message development studies [23], this study omitted a control group because the main purpose was to understand whether health or environmental messages were perceived as more effective relative to each other.” | Lines 175-178 |
| **7** | The presentation of the table requires a lot of work. The presentation of Table 1 is horrible. This single table takes 8 pages with a lot of blank areas on many pages. For table 3, the author can easily add one more column to compare the differences between Health Warning and Environmental Warnings groups as the balance test. As for table 5, there are more professional presentation to report regression results. | We have reformatted all tables to remove extra blank spaces and take up fewer pages. We paid special attention to the formatting of Table 1 and Table 6 (previously Table 5).  For Table 3, we now report balance results between Health and Environmental Warning groups as an additional column (**see also response to Comment #5**).  As for Table 6 (previously Table 5), we considered reporting these results as means instead of coefficients. However, given the number of variables examined, we felt it was easier to see the relationship between each predictor and PME within message type by using coefficients (for example, it is easier to see the linear relationship between increasing age and decreasing PME). | Table 1  Table 3  Table 6 |
|  | **Reviewer #2** |  |  |
| **8** | This study explored participants’ reactions to health and environmental warnings messages about red meat. The manuscript is well written and easy to follow. I have included general and specific notes below on areas of the paper that I believe could be strengthened. | Thank you for your review. Please see specific responses to comments below. | N/A |
| **9** | You mention at the very end of your limitations section that COVID may have elicited higher PME in health warning messages, especially for early death, which seems likely given its salience during the study period. This seems to be a very large limitation of this work, and should be discussed in more detail. Did you collect any data before the nationwide quarantine (i.e., early March)? Is there any way you can do a sensitivity analysis looking at responses from earlier in the pandemic (March) compared to later (April) to see if the effectiveness of the warnings changed over time? In the discussion, I think it would be valid to spend more time reflecting on COVID’s influence on this study. Do you think health warnings will continue to be salient, given the ongoing pandemic? Is there any scientific evidence that you can cite that supports or negates the idea that the salience of health risks created by COVID may have influenced your findings? | Upon closer review of our dataset, all data were collected between March 22-March 25, 2020. Given this, there is not a sufficient difference in time to conduct sensitivity analyses. We have updated the text to indicate that all data were collected in March 2020 (Line 156):  “From March 22-March 25, 2020, we recruited a sample of 1,199 U.S. adults aged 18 years and older using Cloud Research’s Prime Panels…”  Secondly, we would note that the goal of this study was to develop and test messages in the current context, which is an ongoing pandemic. In that sense, given that we are going on our third year of this pandemic, the results of this messaging study in the context of a larger health threat continue to be relevant. However, we have not seen specific research that examines how the pandemic influences reactions to health messages more generally. We have updated the discussion to reflect more on COVID’s potential influence on this study and note that it is important to understand the intersection of COVID-19 and health messaging in the future (Lines 515-521):  “Public health agencies widely communicated the elevated risk of severe COVID-19 infection and death for individuals with pre-existing conditions [24]. Many of these pre-existing conditions are associated with diet, such as type 2 diabetes and cardiovascular disease [25]. Additionally, research has found that the COVID-19 pandemic increased mortality salience, leading to increased health behavior intentions [26, 27]. Future research should examine how the salience of health and mortality risks caused by the COVID-19 pandemic may impact health warning messages.” | Line 156  Lines 515-521 |
| **10** | I think this manuscript could benefit from a more in-depth discussion of the application of red meat warnings. How policy-relevant is this work? Is there any way such warnings could be legally required, either in the US or internationally? Do you foresee these warnings being used in educational materials or PSAs? | We have existing text in the introduction discussing the political feasibility of red meat warnings (Lines 76-83):  One potential population-level strategy is to require warning messages to appear on products containing red meat. These messages could focus on a variety of themes, including both health effects and environmental effects of red meat consumption. Five countries have passed policies requiring nutrient warnings on unhealthy foods [28], and seven U.S. states are considering similar policies for sugary beverages [29]. Research has highlighted the promise of warning messages to reduce tobacco [30, 31], sugary beverages [32, 33], and junk food and alcohol purchases [34]. However, studies have not yet examined health warnings on red meat.  Additionally, we have incorporated the following text into the discussion to expand upon the political relevance of red meat warnings (Lines 492-500):  “This research was conducted with the goal of informing future policies to inform consumers about the harms of red meat and discourage consumption. While warning labels on other food products, such as sugary beverages, have been successful in several countries, policies explicitly focused on red meat have not been implemented, so legal and political feasibility in the U.S. must be considered. In the US, warning labels would may be difficult to implement due to First Amendment challenges and previous legal challenges of sugary beverage warning labels in San Francisco [35]. Furthermore, the strong political power of meat industries [35, 36], as well as deep-rooted ideologies of carnism and neoliberalism [36], could challenge the political feasibility of implementing these policies.” | Lines 76-83  Lines 492-500 |
|  | **Introduction** |  |  |
| **11** | p. 3, line 51: Can you give the reader context for what 284 grams of red meat looks like in a person’s diet, perhaps in servings of steak or burgers? | We have updated the text to indicate that 284 grams of red meat is approximately 2.5-quarter pound hamburger patties.  “On average, U.S. adults consume 284 grams of unprocessed red meat per week [37] (approximately 2.5-quarter pound hamburger patties), nearly three times the recommended level identified in the landmark 2019 EAT-*Lancet* report *Healthy Diets from Sustainable Food Systems* [38].” | Lines 50-53 |
| **12** | p. 3, line 60: typo: “knowledge of a safe consumption levels” | Revision incorporated. | Lines 63-64 |
|  | **Methods** |  |  |
| **13** | General: Could you standardize your language throughout the methods section to have consistent terminology for your main experiment, the behavioral framing task, and the causal variant task? I found it a little confusing when you referred to your main experiment as the “between subjects” experiment, because it appears that all three of the tasks in your study were between subjects (in the second and third tasks, participants were randomized to see only one message, correct?). | We have standardized the language throughout to have consistent terminology for the main experiment, the behavioral framing task, and the causal variant task. | N/A |
| **14** | p. 14, line 122: Can you explain why you tested 8 health messages and 10 environmental messages, instead of 8 and 8? Even just a few additional words at the end of the sentence in lines 122-123 would be helpful. Something like, “we created a final list of eight health messages…to be used in this experiment CHOSEN BASED ON X CRITERIA” | This difference in the number of messages was primarily due to having more options for relevant environmental topics than health topics. We have updated the text to clarify this:  “The number of messages selected for health and environment reflected the number of distinct topics for which we could find scientific support (**Table 1**), as well as discussions with the expert advisory committee.” | Lines 132-134 |
| **15** | p. 14, lines 123-124: You list 9 health harms here (not 8), and mention colon and rectal cancer twice—could you clarify? | We have removed rectal cancer from the list of health harms, as this was not tested separately from colon cancer. We list colon cancer twice, as we tested one health warning label for colon cancer specifically, and we tested another health warning label for colon and rectal cancer. We have updated the list with semicolons to clarify this point:  “The health harms were risk of: diabetes; several types of cancer; colon cancer; colon and rectal cancer; cardiovascular disease; heart damage; stroke; and early death (e.g., “Eating red meat increases your risk of early death”).” | Lines 134-136 |
| **16** | p. 14, line 126: Can you separate your list with semicolons to make it clear that you didn’t accidentally list climate change twice? I had to reread that sentence to understand what you were trying to say. | Revision incorporated.  “Environmental harms were: climate change; climate change, which leads to extreme weather events; global warming; carbon footprint; greenhouse gas emissions; water pollution; water shortages; deforestation; environment; and planet.” | Lines 137-139 |
| **17** | p. 17, line 188: Can you be more specific about your primary outcome, given that you’re assessing PME in three separate tasks? ie, add a note that it’s PME for the health/environment warning messages in the main experiment | Revision incorporated.  “The primary outcome for the main experiment was PME of the health and environmental warning messages.” | Lines 207-208 |
| **18** | p. 19, lines 218-222: While balance tests may be unnecessary for randomized experiments, I still think it’s useful to check for differences in demographics and if one is found, conduct a sensitivity analysis on your primary outcome to ensure it isn’t affecting your results. | We appreciate the suggestion and have conducted sensitivity analyses. These are now reported in **Table 3** and **S1 Table**.  “We used chi-square tests to check that socio-demographic characteristics were balanced across arms (**Table 3**). Of the nine balance tests conducted, 1 was significant: participants randomly assigned to the environmental arm were slightly less likely to report consuming less meat as compared to a year ago (24.8%) relative to participants randomly assigned to the health warning arm (23.4%) (*p*=0.04).  To assess whether PME varied by message type (health vs. environmental) in the main experiment, we used a t-test to compare PME ratings (averaged across all warning messages) between the two conditions. Results did not differ in direction or statistical significance when we used linear regression controlling for the unbalanced covariate (change in red meat consumption) (**S1 Table**), so we retained the unadjusted analysis.” | Lines 240-249  Table 3  S1 Table |
| **19** | p. 19, line 223: “between-subjects’ arms”: see general comment under methods section heading. Could you refer to this as your main experiment (or something like that)? | We have updated the text to clarify the differences between the main experiment, behavioral framing task, and the causal variant task.  “To compare the different messages within topic in the main experiment…”  **See also our response to Comment #13.** | Line 251 |
| **20** | p. 19, line 228: “most discouraging message”: You had previously mentioned asking participants which message was most discouraging under Procedures lines 162-163, but I didn’t see that as an outcome of interest in the measures section. | This information was mistakenly omitted from the Measures section. We added the following text to the manuscript:  “Secondary outcomes for the main experiment were intentions to limit red meat consumption over the next seven days and selection of the most discouraging message in each message category (health and environmental). Intention to reduce red meat consumption in the next seven days [39] was measured using the same five-point Likert scale responses as PME. To measure the most discouraging message, participants were asked “Which of these messages ­most discourages you from wanting to eat red meat?” The answer choices were the eight health or ten environmental warning messages (dependent upon condition).” | Lines 219-225 |
| **21** | p. 19, lines 230-236: How did you determine which predictors to include in your models? | Predictors were chosen based on factors that are known in the literature to influence dietary intake and particularly red meat intake. We added the following sentence and references to the text:  “Predictors were chosen based on characteristics previously found to be associated with food purchases and dietary intake overall and red meat intake in particular [40, 41].” | Lines 262-264 |
|  | **Results** |  |  |
| **22** | Table 3: When you refer to “meat” in this table, is that red meat? If so, can you modify to “frequency of red meat consumption” and “red meat consumption as compared to a year ago”? | Revision incorporated. | Table 3 |
| **23** | pp. 24-25, lines 261-269: Can you list the p-values for the significant findings you’re reporting, beyond just saying they’re <0.05? It’s hard to understand what a significant difference of 0.1 on the PME scale means without added context. | We have updated the text to include the specific p-values for the specific findings.  Health Messages (Lines 296-300):  “After adjusting for multiple comparisons, nine statistically significant differences (*p<*0.05) between specific harms emerged. Early death and colon and rectal cancer messages elicited higher PME ratings than cardiovascular disease (*p*=0.008 and *p*=0.001, respectively). The diabetes message elicited lower PME ratings than all other messages (*p*<0.001 for all 7 comparisons).”  Environmental Messages (Lines 302-306):  “After adjusting for multiple comparisons, there were four statistically significant differences (*p*<0.05). Environment performed better than carbon footprint (*p*=0.001), water shortages (*p*=0.032), and climate change (*p*=0.080). Water pollution performed better than carbon footprint (*p*=0.001).” | Lines 296-300  Lines 302-306 |
| **24** | Table 4: Why was the SD for global warming so small (0.05) compared to the others (~1.2-1.3)? | Thank you for bringing this to our attention. This was a typo. We have replaced the standard deviation for global warming with the proper value (1.24) in **Table 5** (previously **Table 4**). | Table 5 |
| **25** | Figure 3: How was the order of messages on the X axis determined? Could you order them from highest to lowest PME score (and add a note at the bottom of the figure that they’re arranged that way) so that readers can see the relationship between the discouragement outcome vs. PME outcome, given that they’re both trying to predict behavior? | Thank you for this suggestion. We have reorganized the messages on the x-axis from highest to lowest PME score and included the suggested footnote.  We also included an additional supplemental table (**S2 Table**) that shows the p-values comparing each message to each other from the z-test. | Figure 3  S2 Table |
| **26** | p. 29, lines 290-293: There appear to be other significant differences in predictors for PME for the health and environmental message models individually that weren’t commented on in the text (e.g., income, political affiliation, ethnicity)—could you either add those results to the text or provide justification for excluding them? | We have incorporated text to describe the other significant predictors (income, political affiliation, and ethnicity).  “Environmental messages elicited higher PME scores among Hispanic/Latinx participants compared to Not Hispanic/Latinx participants (*p*=0.01); among liberal participants compared to moderate (*p*=0.009) and conservative (*p=*0.001) participants; and among participants with an annual household income of $100,000 or more compared with participants with an annual household income of $24,999 or below (*p*=0.002). Health messages elicited higher PME scores among Black participants compared to White participants (*p<*.05); and among participants with a higher annual household income compared to $24,999 or below, except for $75,000-$99,999.” | Lines 333-340 |
|  | **Discussion** |  |  |
| **27** | General: Your main takeaway for differences between warning messages was that colon and rectal cancer and early death were most effective (based on PME), but based on your discouragement outcome, only early death was “effective”. I’m curious as to why you chose to highlight both outcomes (PME and discouragement) in the results—which do you think is most predictive of actual behavior change? What do we gain by using the other outcome? | Thanks for this thoughtful question. Our discussion focused on mean ratings of PME as the primary outcome given that meta-analytic evidence indicates the usefulness of PME for message development (see also our response to Reviewer 1, Comment #3 above). Although PME was the primary outcome, we also asked participants to select the one harm they found most discouraging because we have found that this exercise can amplify otherwise subtle differences between messages; that is, we posited that asking participants to select the one most discouraging harm might reveal differences between harms that would be harder to detect when examining differences in mean PME ratings between harms.  We now include a new paragraph discussing the discouragement results and the difference between the PME and discouragement results.  “Although PME was the primary outcome of this study given its predictive value [11], we also asked participants to select which message of all the messages would most discourage them from consuming red meat because forcing participants to select one message might amplify subtle differences in reactions to each warning. For health messages, PME ratings were highest for messages about colon and rectal cancer, followed by early death, and several types of cancer, which all had very similar PME ratings. The overall pattern of top messages was the same when participants were forced to select the most discouraging message, though the differences between messages appeared to be amplified. For example, a substantial margin separated the proportion of participants who selected early death as the most discouraging harm (31% of participants) compared to several types of cancer or colon and rectal cancer (selected by 17% and 13% of participants, respectively), despite these harms performing similarly in PME ratings. The different pattern of results could reflect that PME measures two constructs in addition to discouragement (concern and unpleasantness). It could also reflect that forcing a response in the discouragement choice exercise resulted in a greater difference between messages. Overall, the results suggest that messages focused on cancer and especially early death may be particularly promising for eliciting behavior change.”  For the environmental messages, results were largely consistent when participants were rating messages on PME and when participants were selecting the most discouraging message. Specifically, there was little variability in both PME ratings (range of mean PME scores, 2.20 to 2.32) and in the proportion of participants who selected each message as the most discouraging (range of %s, 6% to 15%). This pattern of results suggests that a variety of environment-related messages would perform similarly.” | Lines 428-449 |
| **28** | General: I think it’s worth commenting on the varied phrasing of the messages you tested, given that your health warnings all used “increases your risk of” but the environmental warnings never used the word “risk” and instead used words like “contributes to”, “increases”, and “harms.” Is there reason to believe that the word “risk” contributed to the health warning messages’ success? Could you craft an environmental message that frames the warning as health risks to the environment? This seems worthy of mention and future research, given your findings. | Thank you for this thoughtful comment. It is possible that the use of the word “risk” contributed to the health warning messages’ success. However, this seems unlikely given that in our casual variant exercise, we tested whether there were differences between different ways of framing obesity (Eating red meat…contributes to, is associated with, increases your risk, or increases your chances…of obesity) and found that there were no differences. Still, it is worth noting that we did not examine causal variants for environmental messages, and this would be useful to do in the future. We agree this is worthy of further exploration and have added several lines to the discussion section to recommend future study.  “Additionally, the difference in PME ratings between the health and environmental messages could be due to differences in the types of causal variants used. All of the health warning messages included the phrase “increases your risk of,” while the environmental warnings used “contributes to,” “increases,” and “harms.” On the other hand, the results from our casual variant exercise suggested that the type of causal language used does not influence PME for messages about red meat. More specifically, for one health topic (obesity), we tested three causal variants (e.g., Eating red meat is associated with, increases your risk, or increases your chances of obesity) and found that there were no differences in PME by causal variant. However, we did not test different causal variants for other health harms, nor for any environmental harms, and thus, it would be useful for future research on messages about red meat to explore whether different causal variants are perceived as more or less effective.” | Lines 401-411 |
| **29** | p. 30, lines 307-308: I think it’s worth spending a little more time talking about significant predictors of PME for health and environment warnings, especially given ethnic and income differences that could be of use for future messaging research. | We have incorporated text into the discussion to elaborate upon other significant predictors of PME.  In the summary paragraph of the discussion, we note (Lines 357-360):  “PME from health warnings was higher among Black participants compared to white participants. For environmental warnings, PME was higher among Hispanic/Latinx participants, participants with an annual household income of $100,000 or more, and liberal participants.”  Then later in the discussion, we have more substantial discussion of these predictors, as follows (Lines 450-473):  “With regards to demographic and personal predictors of PME, health warning messages elicited a higher PME among Black compared to White participants. It is possible this pattern was seen because racial inequities persist in chronic diseases such as type 2 diabetes and cardiovascular disease, wherein the prevalence is higher among Black as compared to white adults [42]. This higher message effectiveness may also be because at the population level Black individuals are already reducing their meat consumption more than white individuals [43], and so the Black population may be more receptive than white population to these types of warning messages. If this pattern of results holds in real-world settings, health warning messages on red meat could serve as a tool for reducing racial inequity in chronic diseases.  Political affiliation and belief in human-caused climate change were the strongest predictors of PME in the environmental message model. As we hypothesized, stronger beliefs in human-caused climate change were associated with higher perceived effectiveness of the environmental messages. Again, this finding is consistent with the findings from “Climate Change and the American Diet” [44]. While these findings indicate that those who do not believe that human-caused climate change may be harder to reach with environmental messaging, only 18% of our sample strongly or somewhat *disagreed* with the statement that human-caused climate change is occurring. This finding is consistent with other national polls of belief in climate change that show that the majority of U.S. residents believe in human-caused climate change [45].  Environmental messages elicited higher PME from liberals than from moderates and conservatives. This finding is consistent with climate change literature that has examined concern for climate change based on political belief [46, 47]. This relationship was not significant in the analysis of health messages. Thus, health messages may be perceived as less politically controversial in U.S. society.” | Lines 357-360  Lines 450-473 |
| **30** | p. 30, lines 309-310: Does your study provide evidence that warnings about red meat could help inform consumers about the health and environmental impacts of products? If so, can you explicitly say that, and if not, can you temper this statement? | We have added a reference to this statement to support the point that warnings about red meat could help inform consumers about the health and environmental impact.  “Warnings about red meat could help inform consumers about the health and environmental impacts of these products [48].” | Lines 361-362 |
| **31** | p. 30, lines 349-355: Couldn’t it also be possible that type 2 diabetes had the lowest PME because most people associate it with sugar and carbs, not red meat? You also found differences in the environment messages that seem worth commenting on—why do you think the environment message performed so much better than carbon footprint? This seems useful to mention given that many labels focus on carbon footprints. | We agree that participants may not associate type 2 diabetes with red meat intake. Our previous study found that only 13% of participants were aware that red meat intake was associated with type 2 diabetes [48]. Given this, we have added the following text to the manuscript (Lines 416-418):  “Furthermore, this difference could be due to the fact that the public has overall low awareness about the association between red meat intake and type 2 diabetes [48].”  While many existing environmental labels focus on carbon footprints, the environment message performed much better than the carbon footprint message in our experiment. This difference could be attributed to participant understanding of carbon footprints; research indicates that consumers do not fully understand the meaning of carbon footprints. We have added the following text to the discussion in response to this concern (Lines 419-425):  Within the messages focused on environmental harms, there were also few differences in PME, with little variability between the top rated and lowest rated messages. The exception to this pattern is that the environment message had a statistically significant PME higher than the carbon footprint, water shortage, and climate change. This result is surprising, as many existing environmental warning labels focus on carbon footprints [49]. This difference could be attributed to participant understanding of carbon footprints; research indicates that consumers do not fully understand the meaning of carbon footprints [50, 51]. | Lines 416-425 |
| **32** | p. 32, lines 356-374: Can you also speak to the other significant differences in predictors that you observed in Table 3? You didn’t mention some of these results until now, so I recommend adding them to the results section before they’re mentioned in the discussion. | We have added the results of the significant differences to the Results text.  “Health messages elicited higher PME scores among women than men (*b=*0.22, *p<*0.05). This relationship was not statistically significant for environmental messages. Environmental messages elicited higher PME scores among Hispanic/Latinx participants compared to Not Hispanic/Latinx participants (*p*=0.01); among liberal participants compared to moderate (*p*=0.009) and conservative (*p=*0.001) participants; and among participants with an annual household income of $100,000 or more compared with participants with an annual household income of $24,999 or below (*p*=0.002). Health messages elicited higher PME scores among Black participants compared to White participants (*p<*.05); and among participants with a higher annual household income compared to $24,999 or below, except for $75,000-$99,999.”  **See also our response to Comment #26.** | Lines 332-340 |
| **33** | p. 34, lines 394-398: You didn’t see statistically significant differences in the causal language or eating behavior language experiments, but could that be because you weren’t powered to see significant effects, given that your sample sizes were much smaller per group for those analyses? Can you provide an explanation of what your study was powered to find? If you were underpowered to detect differences in effects for these two experiments, I would recommend tempering your language here and including this as a limitation. | This was an exploratory study without an a priori power calculation. It is possible that lack of statistical difference was due to lack of power. We have added this to the limitations and tempered the language in the results.  “Furthermore, this was an exploratory study without an a priori power calculation. It is possible that there were differences in consumer responses to messages that we were not powered to detect.” | Lines 505-507 |
|  | **Conclusions** |  |  |
| **34** | I don’t think your study found that health and environmental harms could be used in warning messages about red meat—your study created warning messages about red meat that included health and environmental harms, and you found that health warnings appeared to be more effective. | We have restructured the Conclusions section to focus more on the development of the messages rather than the effectiveness of the health warnings.  “Reducing red meat consumption in the United States is critical for human and planetary health. This study developed several evidence-based health and environmental warning messages for red meat. Results of this exploratory study suggest that health warning messages are perceived to be more effective than environmental messages. Nonetheless, the inclusion of environmental waring messages is an opportunity to educate consumers about the environmental harms of consuming red meat. Future work focusing on choice experiments and purchasing tasks will clarify the impact of these messages on meat consumption and related behavioral outcomes.” | Lines 524-530 |

**References**

1.	Baig SA, Byron MJ, Boynton MH, Brewer NT, Ribisl KM. Communicating about cigarette smoke constituents: an experimental comparison of two messaging strategies. J Behav Med. 2017;40(2):352-9. doi: 10.1007/s10865-016-9795-x. PubMed Central PMCID: PMCPMC5334183.

2.	Grummon AH, Hall MG, Taillie LS, Brewer NT. How should sugar-sweetened beverage health warnings be designed? A randomized experiment. Prev Med. 2019;121:158-66. doi: 10.1016/j.ypmed.2019.02.010. PubMed Central PMCID: PMCPMC6520104.

3.	Hall MG, Lazard AJ, Grummon AH, Higgins ICA, Bercholz M, Richter APC, et al. Designing warnings for sugary drinks: A randomized experiment with Latino parents and non-Latino parents. Prev Med. 2021;148:106562. Epub 2021/04/21. doi: 10.1016/j.ypmed.2021.106562. PubMed PMID: 33878350.

4.	Grummon AH, Reimold AE, Hall MG. Influence of the San Francisco, CA, Sugar-Sweetened Beverage Health Warning on Consumer Reactions: Implications for Equity from a Randomized Experiment. J Acad Nutr Diet. 2021. Epub 2021/09/02. doi: 10.1016/j.jand.2021.07.008. PubMed PMID: 34465443.

5.	Taillie LS, Chauvenet C, Grummon AH, Hall MG, Waterlander W, Prestemon CE, et al. Testing front-of-package warnings to discourage red meat consumption: a randomized experiment with US meat consumers. Int J Behav Nutr Phys Act. 2021;18(1):114. Epub 2021/09/09. doi: 10.1186/s12966-021-01178-9. PubMed PMID: 34493289; PubMed Central PMCID: PMCPMC8423585.

6.	Taillie LS, Hall MG, Gomez LF, Higgins I, Bercholz M, Murukutla N, et al. Designing an Effective Front-of-Package Warning Label for Food and Drinks High in Added Sugar, Sodium, or Saturated Fat in Colombia: An Online Experiment. Nutrients. 2020;12(10). Epub 2020/10/18. doi: 10.3390/nu12103124. PubMed PMID: 33066130; PubMed Central PMCID: PMCPMC7601991.

7.	Grummon AH, Hall MG, Mitchell CG, Pulido M, Mendel Sheldon J, Noar SM, et al. Reactions to messages about smoking, vaping and COVID-19: two national experiments. Tob Control. 2020. Epub 2020/11/15. doi: 10.1136/tobaccocontrol-2020-055956. PubMed PMID: 33188150; PubMed Central PMCID: PMCPMC7669534.

8.	Grummon AH, Smith NR, Golden SD, Frerichs L, Taillie LS, Brewer NT. Health Warnings on Sugar-Sweetened Beverages: Simulation of Impacts on Diet and Obesity Among U.S. Adults. Am J Prev Med. 2019;57(6):765-74. Epub 2019/10/22. doi: 10.1016/j.amepre.2019.06.022. PubMed PMID: 31630966; PubMed Central PMCID: PMCPMC6874759.

9.	Taillie LS, Hall MG, Popkin BM, Ng SW, Murukutla N. Experimental Studies of Front-of-Package Nutrient Warning Labels on Sugar-Sweetened Beverages and Ultra-Processed Foods: A Scoping Review. Nutrients. 2020;12(2). Epub 2020/02/27. doi: 10.3390/nu12020569. PubMed PMID: 32098363; PubMed Central PMCID: PMCPMC7071470.

10.	Noar SM, Bell T, Kelley D, Barker J, Yzer M. Perceived Message Effectiveness Measures in Tobacco Education Campaigns: A Systematic Review. Commun Methods Meas. 2018;12(4):295-313. Epub 2018/01/01. doi: 10.1080/19312458.2018.1483017. PubMed PMID: 31428217; PubMed Central PMCID: PMCPMC6699787.

11.	Noar SM, Barker J, Bell T, Yzer M. Does Perceived Message Effectiveness Predict the Actual Effectiveness of Tobacco Education Messages? A Systematic Review and Meta-Analysis. Health Commun. 2020;35(2):148-57. Epub 2018/11/30. doi: 10.1080/10410236.2018.1547675. PubMed PMID: 30482058; PubMed Central PMCID: PMCPMC6538475.

12.	Noar SM, Bell T, Kelley D, Barker J, Yzer M. Perceived message effectiveness measures in tobacco education campaigns: A systematic review. Commun Methods Meas. 2018;12(4):295-313. doi: 10.1080/19312458.2018.1483017. PubMed Central PMCID: PMCPMC6699787.

13.	Harpe SE. How to analyze Likert and other rating scale data. Currents in Pharmacy Teaching and Learning. 2015;7(6):836-50. doi: <https://doi.org/10.1016/j.cptl.2015.08.001>.

14.	Baker BO, Hardyck CD, Petrinovich LF. Weak measurements vs. strong statistics: An empirical critique of S.S. Stevens' proscriptions on statistics. Educational and Psychological Measurement. 1996;26(2):291-300.

15.	Labovitz S. Some Observations on Measurement and Statistics. Social Forces. 1967;46(2):151-60. doi: 10.2307/2574595.

16.	Labovitz S. The Assignment of Numbers to Rank Order Categories. American Sociological Review. 1970;35(3):515-24. doi: 10.2307/2092993.

17.	Glass GV, Peckham PD, Sanders JR. Consequences of Failure to Meet Assumptions Underlying the Fixed Effects Analyses of Variance and Covariance. Review of Educational Research. 1972;42(3):237-88. doi: 10.3102/00346543042003237.

18.	Baggaley AR, Hull AL. The Effect of Nonlinear Transformations on a Likert Scale. Evaluation & the Health Professions. 1983;6(4):483-91. doi: 10.1177/016327878300600408.

19.	Baig SA, Noar SM, Gottfredson NC, Boynton MH, Ribisl KM, Brewer NT. UNC perceived message effectiveness: validation of a brief scale. Ann Behav Med. 2019;53(8):732-42. doi: 10.1093/abm/kay080. PubMed Central PMCID: PMCPMC6636889.

20.	DeVellis RF, Thorpe CT. Scale development: Theory and applications: Sage publications; 2021.

21.	Johnson RB, Christensen L. Educational research: Quantitative, qualitative, and mixed approaches: Sage publications; 2019.

22.	Moher D, Hopewell S, Schulz KF, Montori V, Gotzsche PC, Devereaux PJ, et al. CONSORT 2010 Explanation and Elaboration: Updated guidelines for reporting parallel group randomised trials. J Clin Epidemiol. 2010;63(8):e1-37. Epub 2010/03/30. doi: 10.1016/j.jclinepi.2010.03.004. PubMed PMID: 20346624.

23.	Hall MG, Grummon AH, Maynard OM, Kameny MR, Jenson D, Popkin BM. Causal language in health warning labels and US adults' perception: A randomized experiment. Am J Public Health. 2019;109(10):1429-33. doi: 10.2105/AJPH.2019.305222. PubMed Central PMCID: PMCPMC6727278.

24.	People with Certain Medical Conditions: Centers for Disease Control and Prevention; [updated October 14, 2021December 10, 2021]. Available from: <https://www.cdc.gov/coronavirus/2019-ncov/need-extra-precautions/people-with-medical-conditions.html>.

25.	Collaborators GBDD. Health effects of dietary risks in 195 countries, 1990-2017: a systematic analysis for the Global Burden of Disease Study 2017. Lancet. 2019;393(10184):1958-72. Epub 2019/04/08. doi: 10.1016/S0140-6736(19)30041-8. PubMed PMID: 30954305; PubMed Central PMCID: PMCPMC6899507.

26.	Liu Y, Lv X, Tang Z. The impact of mortality salience on quantified self behavior during the COVID-19 pandemic. Personality and Individual Differences. 2021;180:110972. doi: <https://doi.org/10.1016/j.paid.2021.110972>.

27.	Evers NFG, Greenfield PM, Evers GW. COVID-19 shifts mortality salience, activities, and values in the United States: Big data analysis of online adaptation. Hum Behav Emerg Technol. 2021;3(1):107-26. Epub 2021/04/07. doi: 10.1002/hbe2.251. PubMed PMID: 33821242; PubMed Central PMCID: PMCPMC8013295.

28.	NOURISHING framework | World Cancer Research Fund International.

29.	Pomeranz JL, Mozaffarian D, Micha R. Sugar-Sweetened Beverage Warning Policies in the Broader Legal Context: Health and Safety Warning Laws and the First Amendment. Am J Prev Med. 2020;58(6):783-8. doi: 10.1016/j.amepre.2020.01.006. PubMed Central PMCID: PMCPMC7246175.

30.	Noar SM, Francis DB, Bridges C, Sontag JM, Ribisl KM, Brewer NT. The impact of strengthening cigarette pack warnings: Systematic review of longitudinal observational studies. Soc Sci Med. 2016;164:118-29. doi: 10.1016/j.socscimed.2016.06.011. PubMed Central PMCID: PMCPMC5026824.

31.	Noar SM, Hall MG, Francis DB, Ribisl KM, Pepper JK, Brewer NT. Pictorial cigarette pack warnings: a meta-analysis of experimental studies. Tob Control. 2016;25(3):341-54. doi: 10.1136/tobaccocontrol-2014-051978. PubMed Central PMCID: PMCPMC4636492.

32.	Grummon AH, Hall MG. Sugary drink warnings: A meta-analysis of experimental studies. PLoS Med. 2020;17(5):e1003120. doi: 10.1371/journal.pmed.1003120. PubMed Central PMCID: PMCPMC7239392.

33.	Taillie LS, Reyes M, Colchero MA, Popkin B, Corvalan C. An evaluation of Chile's Law of Food Labeling and Advertising on sugar-sweetened beverage purchases from 2015 to 2017: A before-and-after study. PLoS Med. 2020;17(2):e1003015. Epub 2020/02/12. doi: 10.1371/journal.pmed.1003015. PubMed PMID: 32045424; PubMed Central PMCID: PMCPMC7012389 and LST, MR, CC, and AC declare that they have no competing interests. BP is on the editorial board and otherwise has no competing interests.

34.	Clarke N, Pechey E, Kosite D, Konig LM, Mantzari E, Blackwell AKM, et al. Impact of health warning labels on selection and consumption of food and alcohol products: systematic review with meta-analysis. Health Psychol Rev. 2020:1-24. Epub 2020/06/10. doi: 10.1080/17437199.2020.1780147. PubMed PMID: 32515697.

35.	Wilde P, Pomeranz JL, Lizewski LJ, Ruan M, Mozaffarian D, Zhang FF. Legal Feasibility of US Government Policies to Reduce Cancer Risk by Reducing Intake of Processed Meat. Milbank Q. 2019;97(2):420-48. Epub 2019/04/25. doi: 10.1111/1468-0009.12385. PubMed PMID: 31016816; PubMed Central PMCID: PMCPMC6554508.

36.	Sievert K, Lawrence M, Parker C, Baker P. Understanding the Political Challenge of Red and Processed Meat Reduction for Healthy and Sustainable Food Systems: A Narrative Review of the Literature. Int J Health Policy Manag. 2020. Epub 2020/12/11. doi: 10.34172/ijhpm.2020.238. PubMed PMID: 33300762.

37.	Zeng L, Ruan M, Liu J, Wilde P, Naumova EN, Mozaffarian D, et al. Trends in Processed Meat, Unprocessed Red Meat, Poultry, and Fish Consumption in the United States, 1999-2016. J Acad Nutr Diet. 2019;119(7):1085-98.e12. doi: 10.1016/j.jand.2019.04.004. PubMed Central PMCID: PMCPMC6689198.

38.	Willett W, Rockström J, Loken B, Springmann M, Lang T, Vermeulen S, et al. Food in the Anthropocene: the EAT-Lancet Commission on healthy diets from sustainable food systems. Lancet. 2019;393(10170):447-92. doi: 10.1016/S0140-6736(18)31788-4.

39.	Malek L, Umberger WJ, Goddard E. Committed vs. uncommitted meat eaters: Understanding willingness to change protein consumption. Appetite. 2019;138:115-26. Epub 2019/03/29. doi: 10.1016/j.appet.2019.03.024. PubMed PMID: 30917940.

40.	Frank SM, Jaacks LM, Batis C, Vanderlee L, Taillie LS. Patterns of Red and Processed Meat Consumption across North America: A Nationally Representative Cross-Sectional Comparison of Dietary Recalls from Canada, Mexico, and the United States. Int J Environ Res Public Health. 2021;18(1). Epub 2021/01/21. doi: 10.3390/ijerph18010357. PubMed PMID: 33466518; PubMed Central PMCID: PMCPMC7796493.

41.	Lacko AM, Maselko J, Popkin B, Ng SW. Socio-economic and racial/ethnic disparities in the nutritional quality of packaged food purchases in the USA, 2008-2018. Public Health Nutr. 2021;24(17):5730-42. Epub 2021/01/28. doi: 10.1017/S1368980021000367. PubMed PMID: 33500012; PubMed Central PMCID: PMCPMC8313635.

42.	Cunningham T, Croft J, Liu Y, Lu H, Eke P, Giles W. Vital Signs: Racial Disparities in Age-Specific Mortality Among Blacks or African Americans -- United States, 1999-2015. MMWR Morbidity and Mortality Weekly Report. 2017;66:444-56. doi: <http://dx.doi.org/10.15585/mmwr.mm6617e1>.

43.	Public views about Americans’ eating habits | Pew Research Center.

44.	Leiserowitz A, Ballew M, Rosenthal S, Semaan J. Climate change and the American diet. New Haven, CT: Yale Program on Climate Change Communication, 2020.

45.	U.S. Public Views on Climate and Energy | Pew Research Center.

46.	McCright AM, Dunlap RE. The politicization of climate change and polarization in the american public's views of global warming, 2001–2010. Sociol Q. 2011;52(2):155-94. doi: 10.1111/j.1533-8525.2011.01198.x.

47.	Sintov ND, Abou-Ghalioum V, White LV. The partisan politics of low-carbon transport: Why democrats are more likely to adopt electric vehicles than Republicans in the United States. Energy Research & Social Science. 2020;68:101576. doi: 10.1016/j.erss.2020.101576.

48.	Grummon AH, Goodman D, Jaacks LM, Taillie LS, Chauvenet CA, Salvia MG, et al. Awareness of and reactions to health and environmental harms of red meat among parents in the United States. Public Health Nutr. 2021:1-11. Epub 2021/07/30. doi: 10.1017/S1368980021003098. PubMed PMID: 34321133.

49.	Rondoni A, Grasso S. Consumers behaviour towards carbon footprint labels on food: A review of the literature and discussion of industry implications. Journal of Cleaner Production. 2021;301:127031. doi: <https://doi.org/10.1016/j.jclepro.2021.127031>.

50.	Hartikainen H, Roininen T, Katajajuuri J-M, Pulkkinen H. Finnish consumer perceptions of carbon footprints and carbon labelling of food products. Journal of Cleaner Production. 2014;73:285-93. doi: <https://doi.org/10.1016/j.jclepro.2013.09.018>.

51.	Hammond G. Time to give due weight to the 'carbon footprint' issue. Nature. 2007;445(7125):256-. doi: 10.1038/445256b.
