# Supplementary material for: Developing health and environmental warning messages about red meat: An online experiment
Source: PLoS One. 2022 Jun 24;17(6):e0268121. doi: 10.1371/journal.pone.0268121 (PMC9231779; doi:10.1371/journal.pone.0268121)
Supplement: Attachment — Submitted filename: PLOS One Revision 2 Response Letter_FINAL.docx [file pone.0268121.s005.docx]

March 25^th^, 2022

Dr. Emily Chenette
Editor-in-Chief

PLOS One

Dear Editor,

We thank you and the reviewers for your additional comments on our recently submitted manuscript titled “Developing health and environmental warning messages about red meat: An online experiment” (ID PONE-D-21-09001R1).

We are attaching a revised version of the manuscript with track changes. We are also attaching a table to this letter that contains point-by-point responses to the reviewers’ comments.

We look forward to hearing from you regarding our submission. We would be happy to respond to any further questions and comments that you might have.

Kind regards,

Lindsey Smith Taillie, PhD

Assistant Professor, Department of Nutrition 
University of North Carolina at Chapel Hill

**Ms. Ref. No.: ID** PONE-D-21-09001R1

**Title: “**Developing health and environmental warning messages about red meat: An online experiment”

| **#** | **Reviewers’ comments** | **Revision or Response** | **Line Number** |
| --- | --- | --- | --- |
|  | **General** |  |  |
| **1** | Please review your reference list to ensure that it is complete and correct. If you have cited papers that have been retracted, please include the rationale for doing so in the manuscript text, or remove these references and replace them with relevant current references. Any changes to the reference list should be mentioned in the rebuttal letter that accompanies your revised manuscript. If you need to cite a retracted article, indicate the article’s retracted status in the References list and also include a citation and full reference for the retraction notice. | We have reviewed all references. None of the cited papers have been retracted. | Lines 553-842 |
|  | **Reviewer #2** |  |  |
| **2** | Lines 82-83, 96-97: You say that studies have not yet examined health warnings on red meat or developed warning messages about the environmental impact of red meat, but didn’t the study below do that? I understand that this study under review informed the behavioral study cited below and that study was conducted after this one, but it still was published first so I think you need to acknowledge it. You could change this language to something like, “before conducting this study, no research had examined health warnings…” etc.  Taillie, L.S., Chauvenet, C., Grummon, A.H. et al. Testing front-of-package warnings to discourage red meat consumption: a randomized experiment with US meat consumers. Int J Behav Nutr Phys Act 18, 114 (2021). | Thank you for this suggestion. We have now updated the text to clarify that at the time of conducting this experiment, no studies had developed warning messages for health and environmental harms. We also revised the text to clarify the relationship between this study and the Taillie et al. study.  “Yet, currently there is very little knowledge about warning labels on red meat. Specifically, limited research has compared health and environmental warning messages side by side or identified the most promising health and environmental harms to include in warning messages. We recently published a study comparing two specific health warnings (about cancer and early death) and two specific environmental warnings (about environmental damage and carbon emissions) [27]; here, we describe the exploratory research that led to the development of the specific warnings used in that study.” | Lines 91-97 |
| **3** | Lines 197, 405: you used “casual” instead of “causal”, just double check if that happened anywhere else in the paper. | We have corrected this typo and reviewed the document for other instances of the use of “casual” instead of “causal.” We did not find any additional errors.  “Second, we examined reactions to messages with varying strength in causal language (causal variant task).”  “On the other hand, the results from our causal variant task…” | Lines 195-196  Lines 404-405 |
| **4** | Lines 255-256: You say that you corrected for multiple comparisons using Bonferroni’s method—can you specify whether the p-values presented in the paper are corrected, and if so, which ones? | The p-values from the pairwise comparisons of the predicted means are the corrected values from Bonferroni’s method. We have added the following text to Lines 255-256:  “The reported p-values for these comparisons were produced using Bonferroni’s method.”  Additionally, we highlight the use of the corrected p-values in the Methods section in Lines 294-296 and Lines 301-302:  “After adjusting for multiple comparisons, nine statistically significant differences (*p*<0.05) between specific harms emerged.”  “After adjusting for multiple comparisons, there were three statistically significant differences between environmental harms (*p*<0.05).” | Lines 255-256  Lines 294-296  Lines 301-302 |
| **5** | Figure 3: I previously commented about changing this figure and although you said that you made the change, it does not appear to have changed in the revision you submitted. My original comment stands: I think it would be more useful to reorder the x axes in Panels A and B so that messages are ordered from highest to lowest PME score from left to right. You added a footnote saying that message topics are arranged from highest to lowest PME score, but they don’t appear to be. | Our apologies for this error. We have double checked, and this figure is now correct with the X axes reordered from highest to lowest PME scores as you suggest. | Figure 3 |
| **6** | Discussion: I know your discussion is already quite long, but I think the Taillie et al. 2021 paper cited above deserves some acknowledgement in the discussion, given that you are calling for that work to be done at the end of your paper but it already has been done and published. In that published paper you did not comment on how your findings compared to this paper (because it wasn’t published yet), so it seems important to note in this paper that although you found significant differences in this paper, you did not see those differences play out in an actual behavioral task. I know it was one single task and may not have been properly powered, but I think it is still worth discussing. | We agree with your suggestion and have added the following text to the discussion:  “Although perceived effectiveness is a marker for messages’ potential to change behavior, more research is needed to assess whether red meat warnings influence red meat purchasing and consumption behaviors. To our knowledge, only one study has investigated the impact of health and environmental warnings on outcomes related to red meat purchases [27]. That study found that participants perceived health warnings and combined environmental and health warnings as more effective than environmental warnings. However, the warnings did not affect participants’ selections in a product choice task. Given that previous research has shown that perceived message effectiveness is predictive of behavioral change [35], it is unclear why even the health warnings did not reduce selection of red meat in that study. One possibility is that the behavioral task may not have been properly powered to detect differences [27], or that the simplistic nature of an online choice experiment does not reflect how warnings would influence purchasing decisions in the real world. Future research would be useful to understand whether such warnings would discourage meat purchases in a more realistic food retail environment.” | Lines 505-517 |
